# Clustered Occurrence of Osteitis Condensans Ilii in Patients with Symptomatic Hip Dysplasia

**DOI:** 10.3390/diagnostics13101701

**Published:** 2023-05-11

**Authors:** Maximilian Muellner, Katharina Ziegeler, Torsten Diekhoff, Henryk Haffer, Friederike Schömig, Vincent Justus Leopold, Matthias Pumberger, Friedemann Göhler

**Affiliations:** 1 Center for Musculoskeletal Surgery, Charité—University Medicine Berlin, Corporate Member of Freie Universität Berlin and Humboldt-Universität zu Berlin, Charitéplatz 1, 10117 Berlin, Germany; 2 Department of Radiology, Charité—University Medicine Berlin, Corporate Member of Freie Universität Berlin and Humboldt-Universität zu Berlin, Charitéplatz 1, 10117 Berlin, Germany

**Keywords:** arthroplasty, developmental dysplasia of the hip, low back pain, sacroiliac joint

## Abstract

Background: Osteitis condensans ilii (OCI) is a relatively rare benign disease of the lower anterior sacroiliac joint (SIJ) region that can cause symptoms such as low back pain (LBP), lateral hip pain and nonspecific hip or thigh pain. Its exact pathoetiology remains to be clarified. The aim of this study is to determine the prevalence of OCI in patients with symptomatic developmental dysplasia of the hip (DDH) undergoing periacetabular osteotomy (PAO) to identify potential clustering of OCI in a with altered biomechanics of hip and SIJs. Methods: A retrospective investigation of all patients who underwent periacetabular osteotomy in a tertiary reference hospital from January 2015 to December 2020. Clinical and demographic data were retrieved from the hospital’s internal medical records. Radiographs and magnetic resonance images (MRIs) were reviewed for the presence of OCI. A *t*-test for independent variables was conducted to identify differences between patients with and without OCI. A binary logistic regression model was established to determine the influence of age, sex and body mass index (BMI) on the presence of OCI. Results: The final analysis included 306 patients (81% female). In 21.2% of the patients (f: 22.6%; m: 15.5%), OCI was present. BMI was significantly higher in patients with OCI (23.7 kg/m^2^ vs. 25.0 kg/m^2^; *p* = 0.044). Binary logistic regression revealed that a higher BMI increased the likelihood of sclerosis in typical osteitis condensans locations, OR = 1.104 (95%-CI [1.024, 1.191]), as did female sex, OR = 2.832 (95%-CI [1.091, 7.352]. Conclusions: Our study revealed a considerably higher prevalence of OCI in patients with DDH than in the general population. Furthermore, BMI was shown to have an influence on the occurrence of OCI. These results support the theory that OCI is attributable to altered mechanical loading of the SIJs. Clinicians should be aware that OCI is common in patients with DDH and a potential cause of LBP, lateral hip pain and nonspecific hip or thigh pain.

## 1. Introduction

Low back pain (LBP) is a common phenomenon, with a point prevalence of around 7.5% of the global population, corresponding to a total of over 550 million people in 2017 [1]. LBP has a lifetime prevalence ranging from 75 to 84% and not only reduces the health-related quality of life of affected individuals but is also associated with high socioeconomic costs [2,3,4,5,6]. While LBP can be caused by various conditions such as degenerative changes in the sacroiliac joint (SIJ), inflammatory diseases, higher fat infiltration of the paraspinal muscles and intervertebral disc pathologies, no underlying cause is found in most individuals [7].

**Figure 1:**
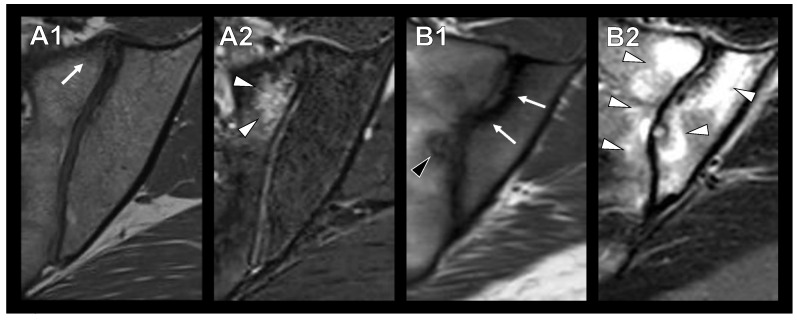
Appearance of OCI and sacroiliitis in axial spondyloarthritis in axial T1-weighted (**A1**,**B1**) and T2-STIR (**A2**,**B2**) MR images. A1/A2: Patient with OCI and anterior sclerosis (**A1**, white arrow) and bone marrow edema (**A2**, white arrowheads). B1/B2: Patient with sacroiliitis with randomly distributed sclerosis (**B1**, white arrows) and bone marrow edema (**B2**, white arrowheads) as well as erosions (**B1**, black arrowhead).

Osteitis condensans ilii (OCI) is a rare cause of LBP that is widely unknown in daily clinical practice [8]. First described by Sicard et al. in 1926 [9], OCI is a clinical condition usually discovered incidentally on radiographs. Most cases of OCI do not cause pain, although some patients suffer from LBP, lateral hip pain, or nonspecific hip or thigh pain [10]. OCI is usually diagnosed on the basis of radiologic findings, presenting as triangular sclerosis of the anterior part of the os ilium, and often bilateral but also unilateral [10,11]. An important differential diagnosis of OCI on magnetic resonance imaging (MRI) is axial spondyloarthritis (axSpa), as osteitis can also be present in variable stages and may partially show edema or fat metaplasia [12]. However, OCI usually presents without erosions, while axSpa shows them along with ankylosis and more randomly distributed edema (Figure 1) [12]. Furthermore, symptomatic developmental dysplasia of the hip (DDH) may cause similar symptoms to OCI, such as lateral hip pain and nonspecific hip or thigh pain [13].

Estimates of the prevalence of OCI in the general population range between 0.9 and 8.9% [14,15]. Females are affected more often than males, especially before age 40 [10]. Although the condition has been known for almost a hundred years, its exact cause remains unclear. While the term osteitis condensans ilii implies an inflammatory process, no pathophysiologic evidence for such a process in OCI has been found so far [10]. Pathologic analysis of biopsy specimens from the SIJ in patients with OCI instead reveals a higher concentric osseus deposition and thickened lamellar bone, while articular cartilage and ligaments appear normal [16]. There are several theories of why OCI occurs; the most common is that it is caused by increased mechanical axial stress as well as bone ischemia due to the compression of the intra-abdominal vessels during pregnancy, leading to the development of the typical sclerosis of OCI [10,15,17]. However, this theory has a weak point, as this type of sclerosis is also seen in females without previous pregnancy, and in males [18,19]. As early as 1959, Jaquline and Arlet associated OCI with abnormalities of the hip, such as hip subluxations or coxa profunda [20]. Furthermore, a recent study by Toyohara et al. showed increased mechanical stress of the SIJ in patients with hip dysplasia [21]. However, OCI has been given little attention in the scientific community in connection with the hip joint.

Due to the overlapping symptoms and the reported influence of hip dysplasia on the mechanical load of the SIJ [21], we conducted a study aimed at gaining a better understanding of these issues and determining whether OCI is more common in patients with symptomatic DDH than in the average population. More generally, we wish to add more evidence about the prevalence of OCI to improve the recognition of this clinically often underreported disease as a potential differential diagnosis in patients presenting with LBP. Clustering of OCI in patients with increased mechanical stress of the SIJ due to DDH would support the theory of increased mechanical stress as an underlying mechanism in the development of OCI. To the best of the authors’ knowledge, no previous study has so far investigated patients with hip joint pathology for possible clustering of OCI.

## 2. Methods

### 2.1. Subjects

**Figure 2:**
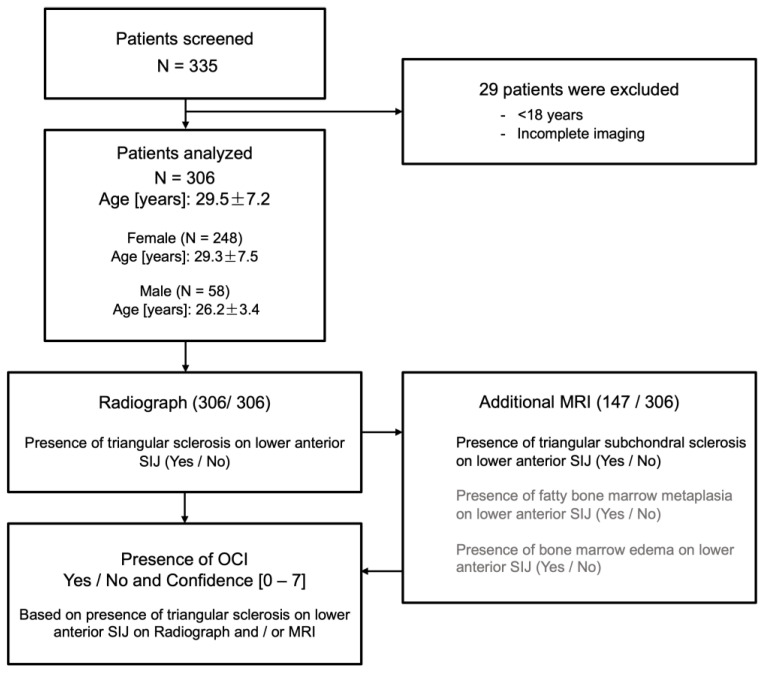
Flow chart of patient selection and radiographic assessment.

The study was approved by the institutional review board (EA4/128/21) and was conducted in accordance with the ethical principles of the Declaration of Helsinki. The investigators adhered to the Strengthening the Reporting of Observational Studies in Epidemiology (STROBE) recommendations. We retrospectively reviewed radiographs and magnetic resonance imaging (MRI) datasets, as well as the medical histories of patients presenting with symptomatic DDH at the authors’ tertiary reference center between January 2015 and December 2020. All patients underwent periacetabular osteotomy (PAO) for symptomatic DDH. As per standard protocol, the imaging workup included an anteroposterior (ap) pelvic overview radiograph and an axial radiograph of the affected hip joint. In addition, each patient underwent a pelvic MRI examination prior to PAO. Patients with incomplete preoperative imaging and younger than 18 years of age were excluded from our analysis (Figure 2). Clinical data such as age, sex, body mass index (BMI) and secondary diseases were collected from the electronic medical records. If patients underwent a contralateral PAO during the study inclusion period, only the imaging and clinical data of the chronologically first intervention were used. The retrospective review of imaging findings for OCI included the preoperative ap pelvic radiographs and MRI datasets (with at least one T1- and one T2-weighted sequence capturing the anterior lower SIJ).

### 2.2. Radiographic Assessment

**Figure 3:**
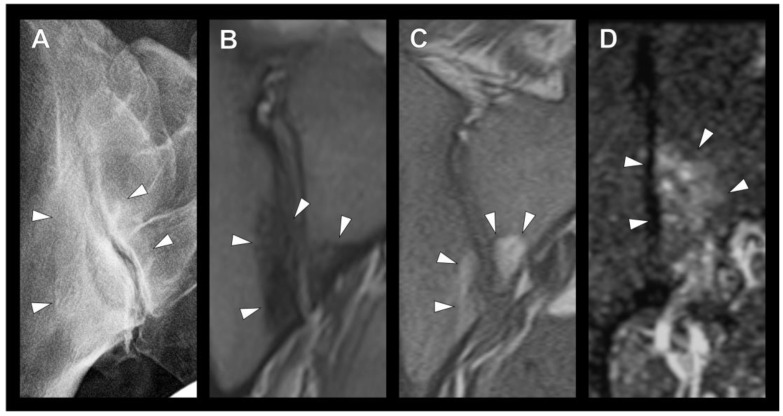
Arrowheads indicate the detected lesion. (**A**) Radiograph of a right sacroiliac joint (SIJ) with osteitis condensans ilii. (**B**–**D**) MR images of the SIJ with the presence of (**B**) sclerosis, (**C**) fatty bone marrow metaplasia and (**D**) bone marrow edema in the typical site of OCI.

Three radiologists (one resident, one board-certified specialist and one consultant) with several years of clinical experience in the evaluation of musculoskeletal images (F.G, T.D., K.Z.) assessed patients’ preoperative radiographs and, if available, MRIs for the presence of OCI/mechanical SIJ stress reaction in the anterior lower joint part (Figure 1). As described in the literature, OCI on radiography was defined as the presence of a triangular sclerosis in the lower anterior part of the SIJ region (Figure 3A) [10]. The MRI was assessed for OCI (triangular subchondral sclerosis of the lower anterior SIJ; Figure 3B), as well as for signs of a mechanical stress reaction such as fatty bone marrow metaplasia (Figure 3C) or bone marrow edema (Figure 3D) in the anterior lower SIJ region. The presence of fatty bone marrow metaplasia or bone marrow edema was not classified as OCI and was not included in further analysis. Furthermore, images were screened for the presence of other (e.g., inflammatory) changes in the SIJ. The group values for the three readers were determined by a two-out-of-three decision on the basis of individual scorings or, if there was no agreement, after discussion in a consensus meeting among the readers. For each rating, the three readers also indicated their confidence (0 to 7, where 0 stands for very uncertain and 7 for absolutely certain), i.e., how certain they were about their rating regarding the presence versus absence of OCI. All disagreements were settled in a consensus meeting of all three raters.

### 2.3. Statistical Analysis

First, the Shapiro–Wilk test was applied to test the continuous variables for normal distribution. For normally distributed continuous variables, the mean and standard deviation (SD) are reported. Categorical variables are presented as total number (N) and percentage (%). Differences in categorical variables were investigated by applying either Chi-square or Fischer’s exact test. A *t*-test for independent variables was used to determine the differences between patients with and without sclerotic lesions in typical locations of OCI. A binary logistic regression model was established with age, sex and BMI set as independent variables to predict the likelihood of sclerosis in typical locations. Inter-rater reliability of adiographic assessment of the SIJ was determined by calculating a mixed two-way intraclass correlation coefficient (ICC) with absolute agreement. The ICC is reported with its 95% confidence interval (CI). All statistical analyses were conducted using SPSS Version 28.0 (IBM Corporation, Armonk, NY, USA), and statistical significance was defined as a *p*-value < 0.05.

## 3. Results

### 3.1. Patients

A total of 306 patients (248 females = 81%) with a mean age of 29.5 years (SD ± 7.2 years) and a mean body mass index (BMI) of 24.0 kg/m^2^ (SD ± 4.0 kg/m^2^) were analyzed. In 53.9% of patients (N = 165), PAO was performed on the right hip. In addition to PAO, a femoral head-neck osteochondroplasty was performed in 33.3% of cases (*n* = 102). Overall, the study population was rather healthy in terms of pre-existing conditions, the most frequent being hypothyroidism in 2.9% (N = 9). The demographic data of our study population are compiled in Table 1. In this population, the overall prevalence of OCI was 21.2% (N = 65); almost one quarter of female patients (22.6%) presented with sclerotic lesions typical for OCI, whereas males were found to have a lower rate of OCI (15.5%). In 21 patients (6.9%), edema was found using MRI.

### 3.2. Sclerotic Lesions in Typical Location of Osteitis Condensans Ilii

Sixty-five of 306 patients (21.2%) presented with a sclerotic lesion in the typical location for OCI, 30 of them with a unilateral and the remaining 35 with bilateral lesions. Females presented with 27 unilateral and 29 bilateral sclerotic lesions. In males, three cases of unilateral and six bilateral sclerotic lesions were detected. Comparing patients with and without OCI-typical sclerosis, we found that patients with sclerosis had a significantly higher BMI (OCI: 25.0 ± 4.4; No OCI: 23.7 ± 3.8; *p* = 0.044). Age and sex did not appear to be significantly different between groups (Table 2). The side of the sclerotic lesion was not associated with the side of PAO.

The binary logistic regression model revealed that BMI, age and sex had a significant influence on the presence of sclerosis in typical locations of osteitis condensans ilii. Of the three variables entered into the logistic regression model, two significantly predicted the presence of sclerosis in these locations: BMI (*p* = 0.010) and sex (*p* = 0.032). A higher BMI increased the likelihood of sclerosis in these locations, OR= 1.104 (95%-CI [1.024, 1.191]), as did female sex, OR = 2.832 (95%-CI [1.091, 7.352] (Table 3).

### 3.3. Imaging Modality and Inter-Rater Reliability

Preoperative radiographs were available for all patients, whereas additional MRI was only available in 147/306 cases (48%). All three raters rated the ap pelvic radiographs for the presence of OCI and, if available, the MRI for the presence of sclerotic lesions, bone marrow edema or fat deposits. Self-reported diagnostic confidence ranged from 4.78 ± 1.25 (rater II) to 6.81 ± 0.54 (rater III), of a maximum score of 7. There was moderate to good inter-rater reliability for diagnosing OCI using the method described here, with an ICC ranging from 0.656 to 0.767 achieved (Table 4).

## 4. Discussion

Although osteitis condensans ilii and its symptoms, such as LBP, have been known for almost a century, its pathoetiology is still controversial. Our study shows a prevalence of 21.6% of sclerotic lesions in typical locations of OCI in patients with symptomatic developmental dysplasia of the hip. Furthermore, we demonstrate that both female sex and BMI significantly increase the odds of developing OCI. Although the condition has been known for about 100 years, relatively few studies have investigated OCI so far. In line with previous studies, more women than men had OCI in our study population (22.6% vs. 15.5%). Overall, the prevalence of OCI was considerably higher than previously reported, although these findings need to be interpreted with caution as we investigated a predominantly female population.

A recent study conducted by Borlandelli et al. demonstrated an OCI prevalence of 1.6% in their female population, consisting of patients who presented to the emergency department and had an ap pelvic radiograph [22]. Although some secondary conditions were investigated, an essential characteristic, namely BMI, was not reported. It is assumed that the mechanical force acting on the SIJ is higher in individuals with an elevated BMI. Furthermore, Borlandelli et al. only report results for female patients because no OCI was present in their male subjects. Eshed and Lidar’s investigation of 289 MRI datasets in patients with suspected inflammatory sacroiliitis revealed OCI in 8.9% of cases [14]. This underlines the importance of OCI as a differential diagnosis in patients with inflammatory low back pain. Unfortunately, the investigators provide no further details apart from frequency. Compared with the results of these most recent studies, we found a considerably higher prevalence of OCI in DDH patients, underlining the importance of hip joint pathologies on the SIJ and conditions occurring with those pathologies.

The pregnancy theory still has wide currency, although OCI also occurs in males and in females without a history of pregnancy. Poddubnyy et al. showed that an average of 7.1 years elapsed between the last birth and the onset of OCI-related complaints, calling into question a causal relationship [23]. Our data revealed that, in addition to female sex, a higher BMI is also more likely linked to the occurrence of OCI. A recent biomechanical study conducted by Toyohara et al. investigated the influence of acetabular dysplasia on SIJ stress [21]. The authors demonstrated an association between acetabular coverage and stress distribution in the SIJ. In patients with higher acetabular coverage, the equivalent stress and compressive stress on SIJ cartilage decreased. These results, together with the distinctively higher prevalence in DDH patients, support the role of altered mechanical loading in the development of OCI. A close relationship between degenerative conditions of the hip and spine was already described by Offerski and MacNab as early as 1983 [24]. Harada et al. recently conducted a study investigating spinopelvic alignment in patients with hip dysplasia and found that patients with this condition had a significantly higher pelvic incidence (PI) than patients without [25]. Abola et al. showed, in a cadaver study, that a high PI was associated with changes in sacral anatomy, which subsequently also affected the sacroiliac joint [26]. It is, therefore, possible that the SIJ load is different in DDH and possibly favors the occurrence of OCI. However, a recent study did not find significant associations between PI and SIJ degeneration in a normal population without LBP [27]. These findings underline the complexity of the dynamic interactions between the spine, pelvis and hips in the lumbopelvic system. During walking—the most common human daily activity—different hip joint forces are generated through the gait cycle [28]. For instance, a recent in-silico study by Ammarullah et al. showed an increase in, and widening of, Tresca stress with increasing BMI in individuals with a hip joint prosthesis [29]. Overall, further investigations of the complex and dynamic lumbopelvic biomechanics are needed to improve our understanding of force-related diseases. In this context, in-silico models could be helpful because of their complexity and could provide fast results in a cost-effective manner. Some of these models have already been established, for instance, for the analysis of hip joint forces [30,31].

As OCI was present in about a quarter of the patients who underwent PAO for symptomatic DDH, we believe that the SIJ should be evaluated more closely before surgery. Since OCI and DDH can cause similar symptoms, it is important to identify the exact cause, as this may guide and educate the patient about OCI and its symptoms. OCI is still poorly understood, which is why no specific therapy is available other than pain relief and physical therapy [10]. Further studies are needed to investigate whether OCI affects the outcome of PAO in patients with DDH.

Among the strengths of our work is that we investigated a rather large number of patients with symptomatic DDH for the presence of OCI. Furthermore, in many of the investigated cases (147/307), two radiologic modalities were used to make an accurate diagnosis of OCI. However, our study is not free of limitations. First, due to our cross-sectional study design, it was not possible to establish causality. The retrospective study design did not allow us to collect all relevant data, such as previous pregnancies, so we could not test the pregnancy theory in our study. These limitations can be overcome in a prospective longitudinal study. Another point to note is that we only studied patients with DDH undergoing surgery; therefore, our results may not be generalizable to all hip dysplasia patients. Moreover, we did not include a healthy control group, which may have resulted in the overrating of OCI. However, two of the three radiologists reading images were certified specialists for musculoskeletal imaging with several years of experience.

## 5. Conclusions

In a patient population with symptomatic DDH, we found a prevalence of OCI of 21.2%, which is considerably higher than in previously reported studies. In addition, a significantly elevated BMI was observed in the subgroup of patients with a sclerotic lesion in the typical location of OCI. These findings, along with what has been reported in the literature, support the role of altered mechanical loading in the development of OCI. Furthermore, our results show that physicians should be aware of OCI as a frequent condition in patients with DDH and a potential cause of LBP, lateral hip pain and nonspecific hip or thigh pain. In particular, patients scheduled for PAO for symptomatic DDH should be more closely evaluated for the presence of OCI as an alternative cause of pain before surgery. To confirm these findings, further investigations with a prospective study design and control groups are needed. Further analysis of lumbopelvic biomechanics should be performed to improve our understanding of force transmission under specific anatomic circumstances, for instance, in an in-silico model.

## Figures and Tables

**Table 1:** Patient demographics. SIJ = sacroiliac joint; OCI = osteitis condensans ilii; PAO = periacetabular osteotomy; PCOS = polycystic ovary syndrome.

	All	Female	Male
N	306	248	58
Age [years]	29.5 ± 7.2	29.3 ± 7.5	26.2 ± 3.4
BMI [kg/m^2^]	24.0 ± 4.0	23.5 ± 4.0	30.5 ± 5.7
Presence of SIJ alterations N (%)			
OCI/Sclerosis	65 (21.2)	56 (22.6)	9 (15.5)
Edema	21 (6.9)	19 (7.7)	2 (3.4)
Local fat deposit	11 (3.6)	11 (4.4)	0 (0)
PAO N (%)			
Right side	165 (53.9)	135 (54.4)	30 (51.7)
Left side	141 (46.1)	113 (45.6)	28 (48.3)
Additional intervention N (%)			
Femoral osteoplasty	102 (33.3)	63 (25.4)	39 (67.2)
Femoral osteotomies	2 (0.6)	1 (0.4)	1 (1.7)
Femoral osteoplasty and osteotomy	1 (0.3)	1 (0.4)	0 (0)
Femoral osteoplasty and surg. hip dislocation	3 (0.9)	0 (0)	3 (5.1)
Comorbidities N (%)			
Asthma	5 (1.6)	5 (2.0)	0 (0)
Diabetes mellitus	1 (0.3)	1 (0.4)	0 (0)
Depression	4 (1.3)	1 (0.4)	3 (5.2)
Anxiety disorders	2 (0.6)	2 (0.8)	0 (0)
Hypertension	2 (0.6)	2 (0.8)	0 (0)
Hypothyroidism	9 (2.9)	8 (3.2)	1 (1.7)
Infantile cerebral palsy	1 (0.3)	1 (0.4)	0 (0)
PCOS	2 (0.6)	2 (0.8)	0 (0)
Migraine	4 (1.3)	4 (1.6)	0 (0)
Meniere’s disease	1 (0.3)	1 (0.4)	0 (0)
Multiple sclerosis	1 (0.3)	1 (0.4)	0 (0)
Other	5 (1.6)	5 (2.0)	0 (0)

**Table 2:** Presence of sclerosis in typical location of osteitis condensans ilii (OCI).

	No OCI/Sclerosis	OCI/Sclerosis	*p*-Value
N = 306	241	65	
Side of sclerosis	n/a	Unilateral: 30; Bilateral: 35	
Age [years]	29.6 ± 7.4	29.0 ± 6.5	0.545
BMI [kg/m^2^]	23.7 ± 3.8	25.0 ± 4.4	0.044
Sex	F:192; M:49	F:56; M:9	0.286
PAO			
Left side	114	27	0.484
Right side	127	38	

**Table 3:** Binary logistic regression model with age, sex and body mass index (BMI) predicting the likelihood of sclerosis in typical locations of osteitis condensans ilii.

		95% CI for Odds Ratio		
Predictors	Odds Ratio	Lower Bound	Upper Bound	*p*
Age [years]	0.980	0.936	1.025	0.379
BMI [kg/m^2^]	1.104	1.024	1.191	0.010
Sex	2.832	1.091	7.352	0.032

**Table 4:** Modality of choice and confidence of the three raters evaluating the sacroiliac joint (SIJ) and inter-rater reliability with 95% confidence interval (CI).

	Used Modality				
Rater	Radiograph	MRI	Both	Confidence	ICC (95% CI)
I	164	35	107	5.48 ± 0.73	0.716 (0.656–0.767)
II	161	27	118	4.78 ± 1.25	
III	157	45	104	6.81 ± 0.54	

## Data Availability

The data presented in this study are available on request from the corresponding author.
